# Integrate Point-Cloud Segmentation with 3D LiDAR Scan-Matching for Mobile Robot Localization and Mapping

**DOI:** 10.3390/s20010237

**Published:** 2019-12-31

**Authors:** Xuyou Li, Shitong Du, Guangchun Li, Haoyu Li

**Affiliations:** College of Automation, Harbin Engineering University, Harbin 150001, China; lixuyou@hrbeu.edu.cn (X.L.); lgc_67@hrbeu.edu.cn (G.L.); 2012071507@hrbeu.edu.cn (H.L.)

**Keywords:** ICP, ground point, dynamic environments, segmentation, closed loops, 6D SLAM

## Abstract

Localization and mapping are key requirements for autonomous mobile systems to perform navigation and interaction tasks. Iterative Closest Point (ICP) is widely applied for LiDAR scan-matching in the robotic community. In addition, the standard ICP algorithm only considers geometric information when iteratively searching for the nearest point. However, ICP individually cannot achieve accurate point-cloud registration performance in challenging environments such as dynamic environments and highways. Moreover, the computation of searching for the closest points is an expensive step in the ICP algorithm, which is limited to meet real-time requirements, especially when dealing with large-scale point-cloud data. In this paper, we propose a segment-based scan-matching framework for six degree-of-freedom pose estimation and mapping. The LiDAR generates a large number of ground points when scanning, but many of these points are useless and increase the burden of subsequent processing. To address this problem, we first apply an image-based ground-point extraction method to filter out noise and ground points. The point cloud after removing the ground points is then segmented into disjoint sets. After this step, a standard point-to-point ICP is applied into to calculate the six degree-of-freedom transformation between consecutive scans. Furthermore, once closed loops are detected in the environment, a 6D graph-optimization algorithm for global relaxation (6D simultaneous localization and mapping (SLAM)) is employed. Experiments based on publicly available KITTI datasets show that our method requires less runtime while at the same time achieves higher pose estimation accuracy compared with the standard ICP method and its variants.

## 1. Introduction

Localization and mapping are crucial tasks for autonomous mobile robot navigation in unknown environments. GPS is one of the widely used solutions for localization, while it suffers from some drawbacks, such as multi-path effect, latency, which limit its application in the city areas and indoor environments [1]. Pose estimation based on inertial navigation systems (INS) and visual sensors has been widely studied over recent decades. INS estimates pose information through integrating acceleration and angular velocity, which are subject to unbounded accumulation errors due to bias and noise from inertial sensors [2]. Vision-based methods can obtain robust and accurate motion estimation; however, they are vulnerable to ambient lighting conditions [3]. As an active sensor, the LiDAR is invariant to light. On the other hand, a typical 3D LiDAR, such as Velodyne VLP-16, can acquire environmental information at around 10 Hz scanning rate with a horizontal field of view (FOV) of 360 degrees and 30(±15) degrees in the vertical direction. High resolution allows the LiDAR to capture a large amount of detailed information in an environment with long ranges. These advantages make LiDAR widely used in robot systems [4].

Point-cloud registration is the basis of the LiDAR-based robot system for localization and mapping. Given two adjacent point-cloud scans with different poses, the goal is to find the transformation that best aligns these two scans [5]. LiDAR-based point-cloud registration methods, also called scan-matching, are generally divided into three categories: point-based methods, feature-based methods and distributions-based methods [6]. The typical point-based method is the iterative closest point (ICP) [7], which iteratively calculates the point correspondences. In each iteration, ICP minimizes a distance function to calculate the transformation between two points clouds according to the selected closest points. Point-to-point ICP uses the point-to-point distance for calculating the closest points, which is the most popular method in the ICP family due to good performance in practice. Many variants of ICP have been proposed (point-to-plane ICP, Generalized-ICP (GICP) [8] for example) to improve the precision, efficiency and robustness of the algorithm.

In addition to point-based methods, scan-matching can also be performed by extracting some low-level attributes in a point cloud. Low-level attributes are geometric features that do not contain semantic information such as normal, intensity, planar surface, edge and some custom descriptor. These methods first automatic extract feature by geometric attributes. Then, feature points are used to find the point correspondences between scans [9]. Lidar Odometry and Mapping (LOAM) achieves a high pose estimation accuracy by extracting edge and plane features [10]. Feature points-based methods find corresponding points by extracting feature points. However, many feature descriptors are designed for applying to specific environmental conditions. Moreover, this method achieves poor estimation accuracy in environments with low geometric information, such as highways [11]. Another category is distribution-based methods. The Normal Distribution Transform(NDT) represents points as a set of Gaussian probability distribution. Instead of working directly on points, this method iteratively calculates point-to-distribution or distribution-to-distribution correspondences and minimizes a distance function in each iteration step [12].

Although there are many excellent point-cloud registration algorithms, the pose estimation suffers from error accumulation in long-term or large-scale scene [13]. A solution is to combine feature-based mapping(e.g., edge-based [14]) with point-based scan-matching algorithm which can limit this accumulation error. Simultaneous localization and mapping (SLAM) method has been shown great success over the past decade [15]. It uses the scan or image data to create a globally consistent representation. Commonly, simultaneous localization and mapping (SLAM) consists of two parts, the frontend and the backend. The frontend involves data association and sensor pose initialization. In the backend either filtering methods or pose-graph-optimization methods are used. This process aims to obtain a globally consistent mapping. Currently, graph-based optimization is the most popular technology in the SLAM field. In a graph-based network, nodes represent robot poses at different locations, and edges correspond to neighbor relations between them [16].

In this work, we propose a segment-based scan-matching framework for six degree-of-freedom pose estimation and mapping. There are four contributions in this paper. First, a 2D image-based ground points extraction method is introduced as a preprocessing step for ICP matching. LiDAR acquires a large number of 3D points while scanning the surrounding scene, which contains many ground points. The computation of the closest points is an expensive step in the standard ICP algorithm, which does not meet real-time requirements. Ground points on flat roads contain little geometric information while the standard point-to-point ICP algorithm only considers the point-to-point Euclidean distance for searching for closest points. Hence, these ground points can cause large corresponding point errors. Furthermore, Ground point extraction is also a key step in point-cloud segmentation. Secondly, point cloud after removing the ground points is then grouped into many clusters. By clustering, some outliers that do not have common attributes are removed. After this step, these different clusters are merged into a new point cloud. Compared to the original point cloud, the ground points of the new point cloud are removed and some false ground points and noise points are also filtered out. This will greatly increase the efficiency and accuracy of ICP matching. Thirdly, we extended the work of the 6D SLAM by combining the segmentation algorithm which has improved the pose estimation accuracy and efficiency with respect to the standard 6D SLAM. On this basis, a systematic evaluation based on urban, country and even highways with both absolute and relative error metrics is presented. The results validate that removing ground points can indeed improve the pose estimation accuracy of ICP and 6D SLAM. It also demonstrates that 6D SLAM performs better in pose optimization for point clouds without ground points with respect to raw point cloud. Furthermore, we also analyzed the possible error sources in different scenarios in detail. In addition, the effective evaluation of standard ICP variants and 6D SLAM in KITTI benchmark enriches the application research of these algorithms which can be considered to be a supplement to the performance of these methods in highly dynamic and complex scenarios.

The remainder of the paper is organized as follows. In Section 2, we summarize related works in ground points extraction, ICP, SLAM and segment-based localization and mapping methods. In Section 3, the proposed algorithm is described in detail. Experimental results are presented in Section 4. The paper ends with discussion in Section 5 and conclusion in Section 6.

## 2. Related Work

There is an increasing body of scholarly work regarding localization and mapping with LiDAR-based method. In this section, we present a brief literature review that is related to our current work.

The point cloud obtained by LiDAR contains many ground points, which poses a challenge to the classification, registration and tracking of subsequent point-cloud processing. Therefore, ground points removal is important in the point-cloud preprocessing step. The typical approach is *Bounding Box Filter* [17]. Points can be excluded from a rectangular bounding region through using this filter. The volume of the box is specified by defining the maximum and minimum coordinate values in the x,y,z directions. For example, in a coordinate system with z-axis up, ground points can be filtered out by setting the appropriate minimum coordinate value of the z-axis. This method is simple and easy to understand but parameters need to be adjusted according to different scenes and where the lidar is installed. Na et al. [18] computed local features with normal and gradient, then ground points were extracted by performing region growing. However, this method increases the computational burden which cannot meet real-time requirements. In [19], a probability occupancy grid-based ground segmentation method is proposed which can run online in different traffic scenarios. Shan et al. [20] projected point cloud onto a range image then extracted ground points by calculating the neighborhood relationship between adjacent scan lines. It is obvious that the neighborhood relationship on the 2D image is easier to calculate. At the same time, operating on 2D images enables a fast segmentation for each scan.

Point-cloud segmentation based on machine learning is also a mature research area. Pomares et al. [21] compared 23 state-of-the-art machine learning-based ground point extraction methods (e.g., SVM and KNN) through the MATLAB Classification Learner App which shows a promising ground extraction accuracy. Hackel et al. [22] developed a supervised learning framework for point-wise semantic classification. Feature descriptors considering neighborhood relationships are input into a random forest classifier, which can accurately and efficiently segment the semantic attributes of the scene, such as ground, cars, and traffic lights. However, traditional machine learning methods rely heavily on hand-crafted feature descriptors. In recent years, deep learning technologies have been applied to the field of 3D point-cloud processing. Velaset et al. [23] segmented the ground and non-ground points by employing a convolutional neural network (CNN) framework. Qi et al. [24] proposed the first deep learning network (PointNet) which directly consumes raw point clouds. PointNet differs from other frameworks in that it only uses fully connected layers to extract features instead of CNNs. Although traditional machine learning or currently popular deep learning frameworks achieves excellent segmentation performance, these supervised learning methods require pre-labeled data sets to train the model. In addition, the GPU must be used to speed up the training process. All these limit the application of learning-based methods.

Iterative closest point (ICP) is the most popular method in point-cloud matching. The most mature and widely used method is the point-to-point ICP method, which uses the point-to-point distance for calculating the closest points [7]. There are also many variants of ICP, such as point-to-plane ICP and GICP [8]. The former uses the point-to-plane distance to search for the closest points, while the latter unifies the point-to-point and point-to-plane iterative closest point algorithms into a probability framework. These two methods need to calculate the tangent plane of each point, while the point-to-point ICP algorithm performs directly on the raw points. Obviously, the point-to-point ICP algorithm is simple and more efficient. Non-geometric information has been also integrated into scan matching to improve the accuracy and efficiency of point-cloud registration. Huhle et al. [25] took color information as an additional dimension on the Normal Distributions. Although this method improves the accuracy of point-cloud registration, the color information is not often included in the raw point-cloud data. Algorithms that only deal with 3D point-cloud coordinates are obviously more general and practical.

In [26], the authors first segmented a single scan into three different semantic categories, i.e., floor, object and ceiling points. After this step, ICP-based transformation was estimated for each individual semantic segment. Since the introduction of semantic information, the corresponding points are only searched within the same semantic category, which greatly improves the possibility of searching for the correct corresponding point while at the same time accelerates the convergence of the ICP. However, the algorithm only uses the gradient relationship between adjacent points to segment the scene, which cannot satisfy complex scenes. In addition, the hand-crafted classifier cannot be extended to outdoor scenes. Inspired by [26], Zaganidis et al. [11,27] integrated semantic information into Normal Distributions Transform (NDT) instead of ICP for point-cloud registration. The method differs from [26] in the semantic segmentation. The method in [27] partitioned the point cloud into sharp edges and planar surface patches according to smoothness while deep learning framework is applied to semantic segmentation in [11]. However, deep learning requires large-scale training data sets, which limits its application in the field of point-cloud registration.

SLAM technology has been widely applied to the robot community in recent years. In the backend, either filter-based methods or pose-graph-optimization methods are used. This process aims to obtain a globally consistent mapping. There are many popular techniques in filter-based methods, such as the Extended Kalman filter [28] and Particle Filters [29]. The differences between these methods mainly focus on sensors, dynamic modes and state-estimation algorithms [30]. However, the main drawback is that the filtering strategy updates probability distributions through time without the convergence guarantee, and suffers from computational complexity or large amounts of particles [31]. In cases where it is difficult to obtain uncertainties and sensor models, these values are often guessed by researchers.

Pose-graph-optimization methods currently have greater advantages in the SLAM over filtering-based methods. Borrmann et al. [32] proposed a 6D SLAM framework that uses ICP to register all scans until convergence. Once closed loops are detected, a GraphSLAM for global relaxation is employed. This algorithm does not require additional point features such as normal, nor does it require high-level features. In [20], a lightweight and real-time six degree-of-freedom pose estimation framework called LeGO-LOAM, is presented. LeGO-LOAM first projects the point cloud into a 2D image. Then, the point cloud is further segmented into the ground and non-ground points. Feature point extraction and matching and error functions are used to estimate six degree-of-freedom pose. In addition, a pose-graph SLAM is also integrated into to obtain more accurate results. LOAM does achieve high pose estimation accuracy at the same time meeting real-time operations. However, feature points-based methods may lead to inaccurate registration and large drift in environments with low geometric information, such as highways.

## 3. Methodology

### 3.1. System Overview

**Figure 1:**
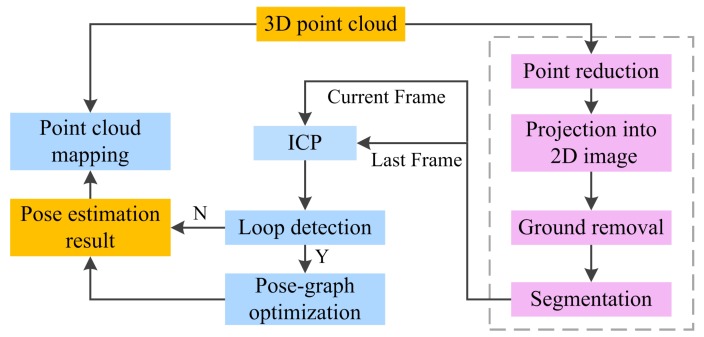
Overview of the proposed LiDAR localization and mapping system architecture.

The architecture of the system is shown in Figure 1, which can be divided into six main modules: point reduction, point-cloud projection, ground points removal, segmentation, ICP and pose-graph optimization (6D SLAM). We first apply an octree-based data structure to reduce the 3D point cloud. An image-based ground point removal method is then introduced. The point cloud after removing the ground point is further segmented into disjoint sets. After this step, a standard point-to-point ICP is applied to calculate the six degree-of-freedom transformation between consecutive scans. In addition, once closed loops are detected in the environment, a 6D graph-optimization algorithm for global relaxation is employed. Our system features a right-handed coordinate system with the z-axis pointing upwards and the x-axis in forward direction. The detailed algorithm principle of each modules will be introduced in the following sections.

### 3.2. Point Reduction

**Figure 2:**
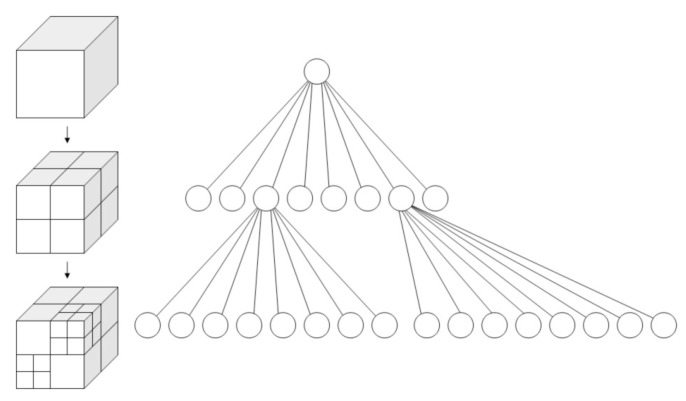
The sparse datastructure of octree.

The high resolution of the LiDAR acquires large-scale data when scanning. For example, Velodyne HDL-64E can generate 1.8 million range measurements per second. Therefore, to process a huge amount of 3D data points efficiently, point-cloud storage and reduction are crucial steps. Octree is a spatial data structure used to describe three-dimensional space which enables efficient storage, compression and search of 3D point cloud. As shown in Figure 2, 3D space is assumed to be a cube and the root node represents a cubic bounding box that stores all points of a point cloud, i.e., 3D coordinates and additional attributes such as reflectance. The octree divides the space into 8 parts, and each node is a part. The sum of the volumes represented by the eight child nodes is equal to the volume of the parent node. In this work, we use an octree-based point-cloud reduction method which is described in detail in [33].

### 3.3. Projection into 2D Range Image

**Figure 3:**
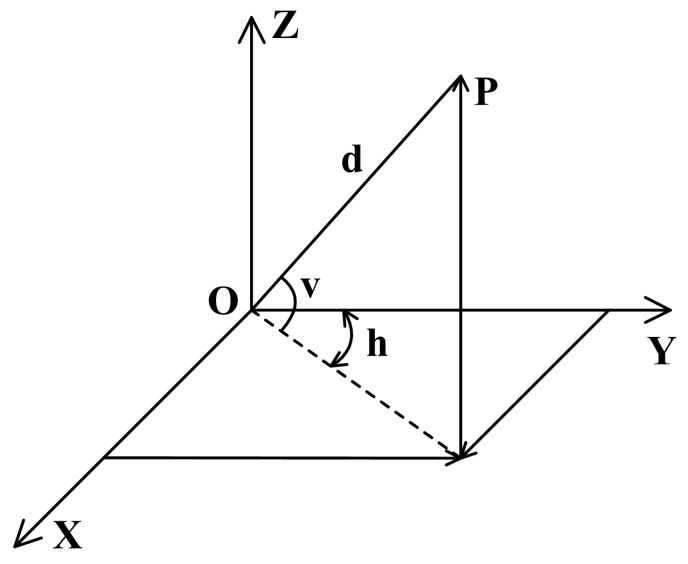
Velodyne LiDAR coordinate system.

Since the subsequent ground points removal and segmentation algorithms are based on 2D range images, we first need to obtain the cylindrical range image. The widely used LiDAR such as the Velodyne family acquires the environmental information by horizontal and vertical scanning. For example, the 16-channel VLP-16 has a horizontal field of view of 360 degrees and 30(±15) degrees for the vertical field of view. If the horizontal azimuth angle $\theta_{h}$ is set to 0.2° and we know from the datasheet that the vertical resolution $\theta_{v}$ is 2°, the corresponding resolution of 2D range image is 1800 by 16. Given a point P=(x,y,z), the corresponding 2D range image is calculated by:

(1) $$\begin{array}{c} \begin{array}{cc} h & =\arctan(\frac{x}{y})*57.3\\ v & =\arctan(\frac{z}{\sqrt{x^{2}+y^{2}}})*57.3\\ r & =\frac{v+v_{b}}{\theta_{v}}\\ c & =\left\lfloor \frac{h-90}{\theta_{h}}\right\rfloor +\frac{h_{s}}{2}\\ d & =I\left(r,c\right)=\sqrt{x^{2}+y^{2}+z^{2}} \end{array} \end{array}$$

where *h* and *v* are the horizontal and vertical angles of *P* in the LiDAR coordinate system, cf. Figure 3. $v_{b}$ represents the maximum vertical field of view of the LiDAR. For the VLP-16, $v_{b}=15$. In addition, $h_{s}=1800$ refers to the horizontal resolution while ⌊⌋ indicates the corresponding number is rounded down. By projecting, each 3D coordinate point *P* is represented by a unique pixel I(r,c) of the image. For the 2D range image, each row of the range image has the same vertical angle $\theta_{v}$, i.e., the same scan lines. Each column indicates points with the same horizontal angle and different scan lines.

### 3.4. Ground Removal

Ground point extraction is a key step in point-cloud processing. In this part, we adopt an image-based ground point extraction method which is similar to [34]. Liu et al. [13] used Equation (2) to extract ground points which is based on an intuitive understanding that the differences in the z-direction between two adjacent points from the same column is much smaller than x and y directions, When the LiDAR scans the ground. However, this assumption is applicable only for ground vehicles. For 3D mobile robots, such as drones, the sensor attitude with respect to the ground must be considered. Moreover, the algorithm traverses points of m rows from the bottom of the image. If $\alpha_{i}$ is smaller than a threshold θ, This corresponding point is considered to be the ground point. However, the user must set different m values and threshold θ according to the installation height of the LiDAR.

(2) $$\alpha_{i}=\arctan\left(\frac{\delta_{z,i}^{c}}{\sqrt{\delta_{x,i}^{c}*\delta_{x,i}^{c}+\delta_{y,i}^{c}*\delta_{y,i}^{c}}}\right)$$

where $\delta_{x,i}^{c}$, $\delta_{y,i}^{c}$, $\delta_{z,i}^{c}$ indicate the differences in x-, y-, and z-direction between two adjacent points from the cth column.

Therefore, in this work, we introduce a more robust and efficient approach. Algorithm 1 depicts the algorithm that we use to extract ground points. First, the 2D range image is converted to an angle image based on Equation (2) (line 2). After conversion, each pixel of the angle image is represented by the corresponding $\alpha_{i}$. Next, a Savitzky–Golay filtering algorithm [35] is applied to the angle image (line 3). This aims to smooth the data and remove noise. After this step, we traverse each pixel from the bottom left of the filtered image. Whenever a non-labeled pixel is encountered, a breadth-first search (BFS) based on the pixel is carried out (line 7–15). The basic idea is BFS starts from the pixel, and find 4 neighborhood from the up, down, left, and right pixels. If the difference between the pixel and its 4 neighborhoods falls into the threshold γ, the pixel is added to the queue, i.e., it is assigned to the ground point (line 12–15). Please note that Label=1 refers to the ground point class. This procedure stops utile the whole connected component receives the same label. Intuitively, this algorithm starts from the bottom left of the image which is generally considered to be a ground point. We assign a label to this point (line 11). BFS is then employed to continuously expand the search until all points belonging to the same label (Label=1) are found. This algorithm traverses all points of the entire image, hence we do not have to manually select m for different hardware platforms.
**Algorithm 1** Ground points extraction**Input:** Range image *R*, ground angle threshold γ, Label=1, windowsize**Output:** L 1:**function**Groundpointextraction(R,Label,L,γ,windowsize ) 2:    A=CreateAngleImage(*R*) 3:    S=ApplySavitskyGolaySmoothing(A,windowsize) 4:    L=zeros($R_{rows}\times R_{cols}$) 5:    **for** r=$S_{rows}$; r ≥ 1; r−− **do** 6:        **for** $c=1;c\leq S_{cols};c++$ **do** 7:           **if** L(r,c)=0 **then** 8:               queue.push(r,c) 9:               **while** queue is not empty **do**10:                   r,c=queue.top()11:                   L(r,c)=Label12:                   **for** $\left(r_{n},c_{n}\right)\subset Neighborhood\left(r,c\right)$ **do**13:                       g=$S\left(r,c\right)-S\left(r_{n},c_{n}\right)$14:                       **if** fabs(g)<γ **then**15:                          queue.push($r_{n},c_{n}$)16:                       **end if**17:                   **end for**18:                   queue.pop()19:               **end while**20:           **end if**21:        **end for**22:    **end for**23:**end function**

### 3.5. Segmentation

**Figure 4:**
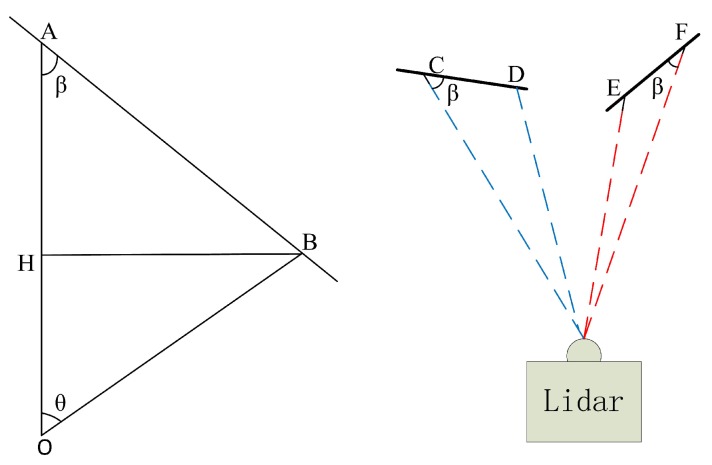
Left: O represents the center of the LiDAR while OA and OB are two laser beams that also represent the distance between the obstacle and the laser sensor. If β>ϵ, where ϵ is a threshold, the two points are considered to be the same cluster. Right: An intuitive example which illustrates the relationship between the β and whether the two points belong to the same object. The blue dotted line is an example that shows C and D belong to the same object and β is larger than the angle in the red dotted line where E and F are from two different objects.

To further remove noise points and outliers, we use the algorithm in [34] to segment the range image after removing the ground point. The idea of this algorithm is similar to the ground points extraction. The method of deciding whether points belong to the same label is shown in Figure 4. As right figure in Figure 4 depicts, β can be used to segment the point cloud if the appropriate threshold ϵ is set. we assume the one with a relatively long distance between OA and OB is $d_{1}$ (∥OA∥) and the other is $d_{2}$ (∥OB∥), then, β is calculated:

(3) $$\beta =\arctan\frac{∥BH∥}{∥HA∥}=\arctan\frac{d_{2}\sin\theta }{d_{1}-d_{2}\cos\theta }$$

where θ is the horizontal azimuth angle or vertical resolution which is described in Section 3.3.

The pseudocode of the algorithm is presented in Algorithm 2. The algorithm differs from Algorithm 1 in input images, the criteria for classification, and the number of labels. $R^{ng}$ represents the image which is directly projected by the point cloud but does not include the ground points. Since the ground point is a category, Algorithm 1 has only one label. However, the segmentation includes many categories. Therefore, the label is automatically incremented by 1 when a cluster is completed.
**Algorithm 2** Segmentation**Input:** Range image $R^{ng}$, segmentation threshold ϵ, Label=1**Output:** L 1:**function**Labelrangeimage($R^{ng}$,Label,L,ϵ) 2:    L=zeros($R_{rows}^{ng}\times R_{cols}^{ng}$) 3:    **for** $r=1;r\leq R_{rows}^{ng};r++$ **do** 4:        **for** $c=1;c\leq R_{cols}^{ng};c++$ **do** 5:           **if** L(r,c)=0 **then** 6:               queue.push(r,c) 7:               **while** queue is not empty **do** 8:                   (r,c)=queue.top() 9:                   L(r,c)=Label10:                   **for** $\left(r_{n},c_{n}\right)\subset Neighborhood\left(r,c\right)$ **do**11:                       $d_{1}=\max\left(R^{ng}\left(r,c\right),R^{ng}\left(r_{n},c_{n}\right)\right)$12:                       $d_{2}=\min\left(R^{ng}\left(r,c\right),R^{ng}\left(r_{n},c_{n}\right)\right)$13:                       **if** $\arctan\frac{d_{2}\sin\alpha }{d_{1}-d_{2}\cos\alpha }>\epsilon$ **then**14:                          queue.push($r_{n},c_{n}$)15:                       **end if**16:                   **end for**17:                   queue.pop()18:               **end while**19:               Label=Label + 120:           **end if**21:        **end for**22:    **end for**23:**end function**

Please note that after the segmentation algorithm is implemented, the 2D image grouped into many sub-images can be easily converted into sub-segments which are represented by 3D coordinate points. We aim to use the segmentation algorithm to remove noise and outliers. Therefore, these different clusters are then merged into a new point cloud. Compared to the original point cloud, the ground point of the new point cloud is removed and some noise and outlier points have also been filtered out. Finally, a standard point-to-point ICP algorithm is then applied to calculate the six degree-of-freedom transformation between consecutive scans. The specific calculation process will be described in the next section.

### 3.6. ICP and 6D SLAM

In this part, point-to-point ICP and a globally consistent scan-matching algorithm are used to calculate six degree-of-freedom pose. In addition, we also compared our result with the standard point-to-planar ICP method and *Bounding Box Filter*-based point-to-point ICP that first removes the ground point by *Bounding Box Filter* and then performs ICP algorithm. The concept of ICP is simple: given an initial guess, it calculates the point correspondences iteratively. Please note that an initial guess is not strictly needed when performing ICP-based scan-matching for LiDAR-based odometry. In fact, the ICP algorithm can be run assuming that the initial rotation and translation are zero as soon as the sensor dynamics is not too fast with respect to the frame rate. In each iteration, ICP minimizes a distance function to calculate the transformation between two points clouds according to the selected closest points. The distance function of point-to-point ICP is defined as:

(4) $$E\left(R,t\right)=\sum_{i=1}^{N_{m}}\sum_{j=1}^{N_{d}}{∥s_{i}-\left(Rd_{j}+t\right)∥}^{2}$$

where $N_{m}$ and $N_{d}$ are the number of points in the source point cloud S and target point cloud D.

Point-to-plane ICP minimizes the sum of the squares of the distances between the source points and the tangent plane of the target points. This specific formula is as follows:

(5) $$T_{opt}=\arg\min_{T}\sum_{i=1}^{N}{\left(\left(Ts_{i}-d_{i}\right)n_{i}\right)}^{2}$$

where *N* is the number of points, and $n_{i}$ is the normal vector corresponding to the target point. T is the rigid transformation between the source and the target points. Compared with the point-to-point ICP, point-to-plane ICP calculates the tangent plane of the point. Therefore, it can achieve better results in environments with low geometric information. However, it needs to calculate the normal vector, which will reduce the efficiency. Hence, point-to-point ICP is used in this work.

ICP obtains a trajectory by calculating the pose between two adjacent scans and then constantly updating it. However, the pose estimation suffers from error accumulation in the long-term or large-scale scene. To address this issue, the pose estimation result of the ICP is input into the 6D SLAM framework, i.e., globally consistent scan-matching [32], once closed loops are detected. It is available in *3DTK-The 3D Toolkit* [36]. 6D SLAM is similar to the point-to-point ICP method but taking into account all scans instead of only two adjacent scans. It solves for all poses at the same time and iterates like in the original ICP. It is actually a pose-graph-optimization method and uses the Mahalanobis distance to represent the global error of all poses. The specific formula is:

(6) $$\begin{array}{c} \begin{array}{cc} W & =\sum_{j\to k}{\left({\bar{E}}_{j,k}-E_{j,k}\right)}^{T}C_{j,k}^{-1}\left({\bar{E}}_{j,k}-E_{j,k}\right)\\ \; & =\sum_{j\to k}{\left({\bar{E}}_{j,k}-\left(X_{j}-X_{k}\right)\right)}^{T}C_{j,k}^{-1}\left({\bar{E}}_{j,k}-\left(X_{j}-X_{k}\right)\right) \end{array} \end{array}$$

where j and k represent scans of the SLAM graph, $E_{j,k}$ is the linearized error metric and $\left({\bar{E}}_{j,k},C_{j,k}\right)$ is the Gaussian distribution. $X_{j}$ and $X_{k}$ are two connected nodes in the graph which represent the corresponding poses. we give only a brief overview here and a detailed description is given in [32].

## 4. Experimental Results

### 4.1. Experimental Platform and Evaluation Method

To evaluate the performance of the proposed algorithm, we test our method in the KITTI benchmark [37]. The datasets are acquired with a vehicle equipped with a Velodyne HDL-64E laser scanner, stereo color video cameras and a high accuracy GPS/INS for ground truth. It contains 11 sequences training data sets, which provide ground truth and 11 test data sets without ground truth. These sequences include three types of environments: urban with buildings around, the country on small roads with vegetations in the scene, and the highway where roads are wide, and the vehicle speed is fast. The HDL-64E has a horizontal FOV of 360° and 26.9° Vertical FOV with 64 Channels whose range reaches 120 m. All data in our experiments are processed on a desktop computer with an i7-7700 3.60 GHz CPU and both algorithms are implemented in C++ and executed in Ubuntu Linux.

The proposed method is evaluated using the absolute metric proposed in [38] and KITTI metric [37], respectively. The absolute metric computes absolute root-mean-square error (RMSE) of translation rotation errors according to Equation (7) to (11)

(7) $$\begin{array}{c} \Delta T_{abs,i}=\left(\begin{array}{cc} \Delta R_{abs,i} & \Delta t_{abs,i}\\ 0 & 1 \end{array}\right)=T_{r,i}T_{e,i}^{-1}, \end{array}$$

where $T_{r,i}$ and $T_{e,i}$ represent the pose matrices of ground truth and estimated pose, respectively in ith frame. Furthermore, the absolute translation error $e_{abs,i}$ and rotation error $\Delta \theta_{abs,i}$ are computed by Equation (8) and Equation (9), respectively.

(8) $$\begin{array}{c} e_{abs,i}=∥\Delta t_{abs,i}∥ \end{array}$$

(9) $$\begin{array}{c} \Delta \theta_{abs,i}=f_{\theta }\left(\Delta R_{abs,i}\right), \end{array}$$

where ∥·∥ indicates Euclidean metric. Then the root-mean-square error(RMSE) of absolute translation errors and absolute rotation errors are calculated by

(10) $$\begin{array}{c} \sigma_{t}=\sqrt{\frac{1}{n+1}\sum_{i=0}^{n}e_{abs,i}^{2}} \end{array}$$

and

(11) $$\begin{array}{c} \sigma_{r}=\sqrt{\frac{1}{n+1}\sum_{i=0}^{n}\Delta \theta_{abs,i}^{2}} \end{array}$$

### 4.2. Results

In this section, we analyze the results of four modules including ground point removal, segmentation, ICP and 6D SLAM. To test the robustness and accuracy of the proposed method to different scenarios, the results of four typical data sequences including urban with buildings around, the country on small roads with vegetations in the scene and a highway where roads are wide, and the vehicle speed is fast are presented.

#### 4.2.1. Ground Points Removal

We compared *Bounding Box Filter* with the ground point extraction method used in this paper, i.e., Algorithm 1. For *Bounding Box Filter*, points can be excluded by designing a rectangular bounding region. The box is specified by defining the maximum and minimum coordinate values in the x,y,z directions. Ground points can be filtered out by setting the appropriate minimum coordinate value of z-axis. According to the installation height and range of the Velodyne HDL-64E laser scanner, the box is set as:

(12) $$\begin{array}{c} \begin{array}{cc} \; & -120<x<120\\ \; & -120<y<120\\ \; & -1.1<z<120 \end{array} \end{array}$$

where x,y,z refer to 3D point coordinates and the unit is the meter.

**Figure 5:**
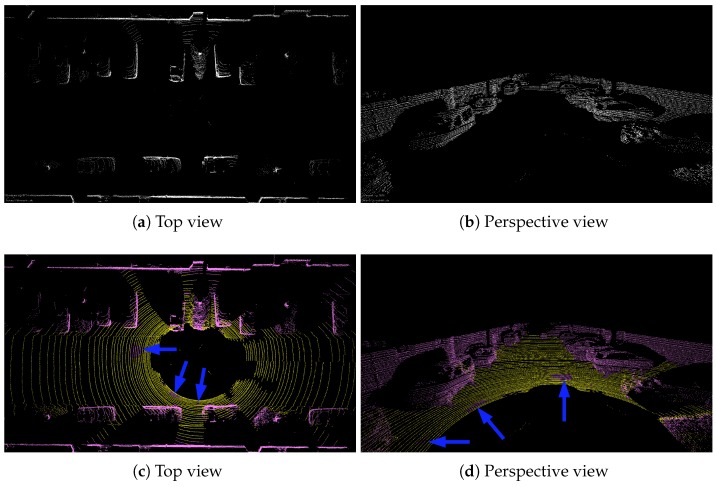
Comparison between *Bounding Box Filter*-based method and the algorithm used in this paper. The above images are a certain frame point cloud of sequence 07 which is collected on the urban. (**a**) ground removal results using the box filtering method from a bird’s eye view. (**b**) the corresponding perspective views. Please note that only non-ground points are displayed in (**a**,**b**). (**c**,**d**) are the results from our method. The yellow part indicates the ground point and the pink color are non-ground points.

**Figure 6:**
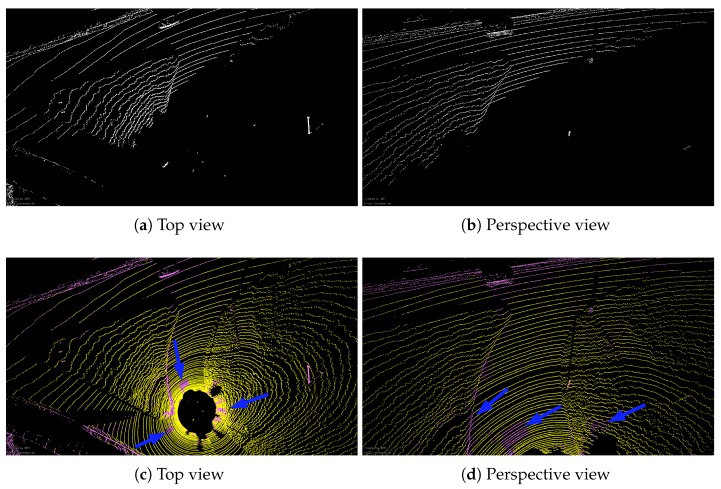
The same as Figure 5, but these images are from sequence 01 which is acquired on a highway.

As for Algorithm 1, ground angle threshold γ and windowsize are set to 5 degrees and 7, respectively. Here, we only qualitatively compare the accuracy of ground point extraction. Two scenarios, including the urban and the highway, are selected to test our algorithm. Please note that Figure 5a,b are the visual inspection from *Bounding Box Filter*, where only non-ground points are presented. For our method, i.e., Figure 5c,d, ground points and non-ground points are displayed in different colors, where the yellow part indicates the ground point and the pink is non-ground points. As shown in Figure 5, two methods have achieved similar accuracy. However, when the same parameters of *Bounding Box Filter* are applied in sequence 01, a large number of ground points are not removed cf. Figure 6a,b.

**Figure 7:**
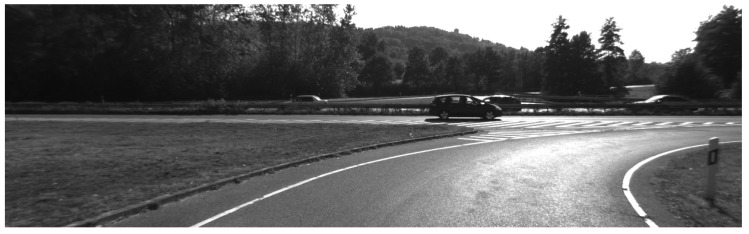
A photo showing the scene corresponding to the point clouds seen in Figure 6.

To help identify ground points, the corresponding real scene is shown in Figure 7. If we want to use the box filtering method to remove all the ground points of Figure 6, the parameters must be changed. Instead, our method achieves the desired results with the same threshold, although some ground points have not been completely removed (blue arrows in Figure 5c,d and Figure 6c,d). The next section will show that these outliers will be removed after using segmentation.

#### 4.2.2. Segmentation

**Figure 8:**
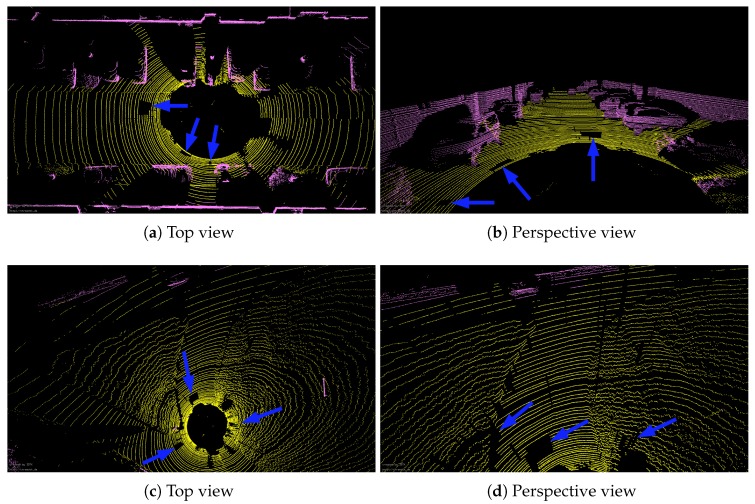
Some results come from the new point cloud, which is merged by different clusters. (**a**) The visual inspection of sequence 07 from a bird’s eye view. (**b**) The visual inspection of sequence 07 from perspective views. (**c**,**d**) are the corresponding results from se01.

To further remove noise points and outliers, we use the method in [34] to segment the range image after removing the ground point. Please note that after the segmentation algorithm is implemented, the 2D image grouped into many sub-images can be easily converted into sub-segments which are represented by 3D coordinate points. By using the segmentation algorithm, those points with the same attributes are assigned to the same labels and the entire point cloud is divided into many sub-segments. We aim to use the segmentation algorithm to remove noise and outliers. Therefore, these different clusters are then merged into a new point cloud. The clusters with fewer than 30 points will be discarded which are most likely to be noise and outliers. Figure 8 shows visual results after running segmentation algorithm. Compared to Figure 5c,d and Figure 6c,d, the ground points of the new point cloud are removed and some false ground points (blue arrows) have also been filtered out.

#### 4.2.3. Comparison of Trajectory Results

In this part, four different scenarios from the KITTI dataset are selected to test the robustness, accuracy and efficiency of the proposed method. We compare the proposed method (SE+PTP) with the standard point-to-point ICP algorithm (PTP), the *Bounding Box Filter*-based ICP method (BBF+PTP), and the point-to-surface ICP method (PTS). Here, BBF+PTP-based method refers to a method that first uses Bounding Box Filter to remove ground points which is then input a standard point-to-point ICP framework. Furthermore, once closed loops are detected, 6D SLAM is used to improve pose estimation accuracy.

**Figure 9:**
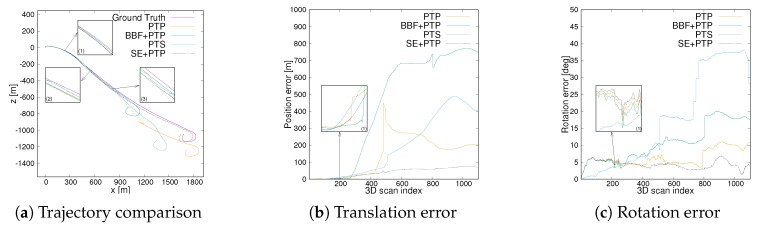
Trajectory and translation as well as rotational error comparison of seq01. (**a**) the trajectory comparison between different ICP. (**b**) the translation error. (**c**) the rotational error.

**Figure 10:**
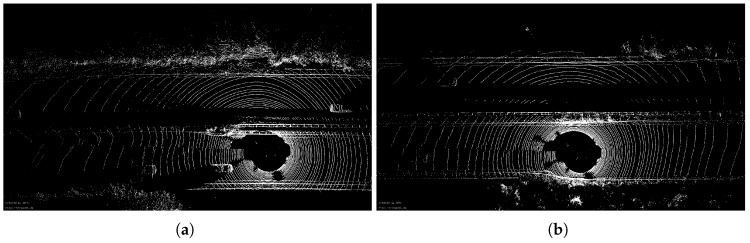
(**a**) The visual inspection corresponding to the Figure 9a(2). (**b**) The visual inspection corresponding to the Figure 9a(3).

Figure 9 compares the 2D trajectory and 3D absolute translation and rotation error of the sequence 01 which is collected on the motorway. As Figure 9a(1) shows, SE+PTP achieves similar performance to BBF+PTP on the first part of the sequence and is slightly better than PTP and PTS. This shows that ICP can find the correct corresponding points with higher probability by removing ground points. On the second part, i.e., Figure 9a(2), SE+ICP is inferior to others but keep similar performance to PTP and PTS. Figure 10a shows the visual inspection corresponding to the Figure 9a(2). PTP and PTS exhibits low-precision in Figure 9a(3) while SE+PTP still maintained within a certain accuracy which can also be seen from Figure 9b(1). Figure 10b shows an example of a point cloud corresponding to Figure 9a(3). Figure 10b contains less geometric and semantic information relative to Figure 10a. This causes PTP and PTS to fail here. Although BBF+PTP does not suffer from large errors here, it finally failed to estimate the pose due to the lack of geometric and semantic information which caused the BBF+PTP-based algorithm to think that the vehicle stayed in place without moving. In contrast, SE+PTP is more robust, which is mainly due to the introduction of the segmentation algorithm. However, our method still cannot accurately estimate the pose of se01. Because there are too many moving vehicles running with high speed.

The absolute translation and rotation error of corresponding sequences to ground truth are given in Table 1, which shows SE+PTP is superior to other methods. An intuitive conclusion is drawn from Table 1 is both BBF+ICP and SE+ICP have improved the accuracy of pose estimation relative to the standard ICP method. This is the result that the segmentation algorithm removes those ground points and noise points. Table 1 also demonstrates the performance of 6D SLAM in different scenarios. 6D SLAM does improve the accuracy of point-to-point ICP alone, cf. PTP and PTP+6DSLAM of se09 in Table 1. The reason is 6D SLAM taking into account all scans instead of only two adjacent scans which limits this accumulation error. Although the position accuracy of PTP+6DSLAM in se14 is similar to PTP, the rotation error has been eliminated. However, PTP+6DSLAM shows worse results than PTP in the urban scene (se07). This is because se07 contains a lot of dynamic vehicles which can cause larger error. The performance of standard 6D SLAM may degrade in a high dynamic environment. In contrast, since SE+PTP+6DSLAM includes a segmentation algorithm, which removes the noise points caused by dynamic objects to a certain extent. Consequently, SE + 6DSLAM achieves excellent results. Another issue that must be noted is the performance of PTS+6DSLAM degrades compared to PTS. This problem is caused by the 6DSLAM algorithm itself. Since 6D SLAM is similar to the point-to-point ICP method but taking into account all scans instead of only two adjacent scans. It solves for all poses at the same time and iterates as in the original ICP. Hence, 6D SLAM is more suitable for point-to-point ICP.

In addition, we also compared the execution time of the programs in Table 2. Compared with PTP (point-to-point ICP), point-to-plane ICP (PTS) needs to calculate the normal vector, which increases the computational. In addition, SE+PTP largely reduces the calculation time compared to standard point-to-point ICP (PTP) due to the ground point removal. For se01, although SE+PTP takes more time than BBF+ICP, cf. se01, the accuracy is much higher. In summary, this experiment of se01 shows that the proposed method can assist ICP to estimate the pose more accurately and efficiently in an environment with low geometric information.

**Figure 11:**
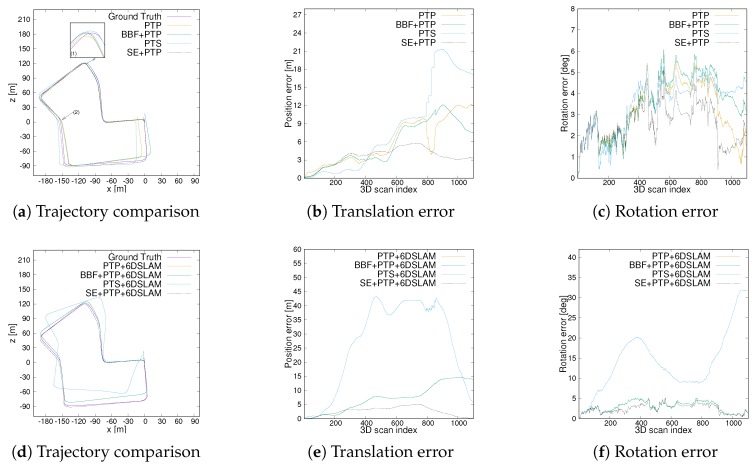
Trajectory and translation as well as rotational error comparison of seq07. (**a**) the trajectory comparison between different ICP. (**b**,**c**) are the translation error and rotational error from ICP. (**d**–**f**) are the corresponding results after applying 6D SLAM.

**Figure 12:**
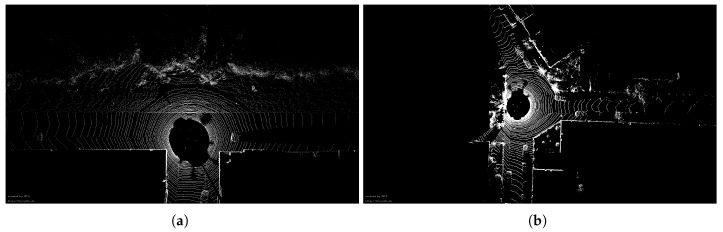
(**a**) The visual inspection of (a)(1) in Figure 11. (**b**) The visual inspection of (a)(2) in Figure 11.

Figure 11 compares the trajectory error from an urban scene. The first row is some results without using 6D SLAM. Overall, the start and end positions of the trajectory from SE-PTP are perfectly coincident, while other methods suffer from significant accumulative errors, cf. Figure 11a. As Figure 11b,c depicts, from the starting point to scan325, PTS presents smaller translation and rotation error than other methods. However, at scan325 (Figure 11a(1)), which is a crossroad, the accuracy of PTS drops rapidly. Starting from scan560, which corresponds to arrow 2 in Figure 11a, the error of BBF+PTP and PTP increases rapidly. In contrast, the error produced by SE+PTP has not changed significantly. Figure 12 are the visual inspections corresponding to the Figure 11a(1,2) which shows that the big error at the corner is caused by the lack of geometric information and the existence of many dynamic objects. As Table 1 shows, SE+PTP achieves better performance compared with PTP, BBF+PTP and PTS, while PTS has larger error with respect to other methods. This shows that point-to-point ICP is more suitable for urban environments, and removing ground points can indeed improve estimation accuracy and efficiency (se07 in Table 2).

After using the 6D SLAM, the trajectory has changed significantly, cf Table 1. First, PTP+6DSLAM and PTS+6DSLAM fail to estimate pose. This is because se07 contains a lot of dynamic vehicles which eventually leads to the performance degradation of the standard 6D SLAM. In contrast, since SE+PTP+6DSLAM includes a segmentation algorithm, which removes the noise points caused by dynamic objects to a certain extent. As a consequence, SE+PTP+6DSLAM achieves excellent results. The result of BBF+PTP+6DSLAM is slightly worse than before but better than PTP+6DSLAM and PTS+6DSLAM, which shows removing ground points helps the convergence of 6D SLAM. Another issue that must be noted is PTS+6DSLAM obtains the same result as PTP+6DSLAM, cf. Figure 11d and Table 1. This shows that 6D SLAM is designed for point-to-point ICP. Overall, compared with other methods, our method requires the less time and achieves higher accuracy.

**Figure 13:**
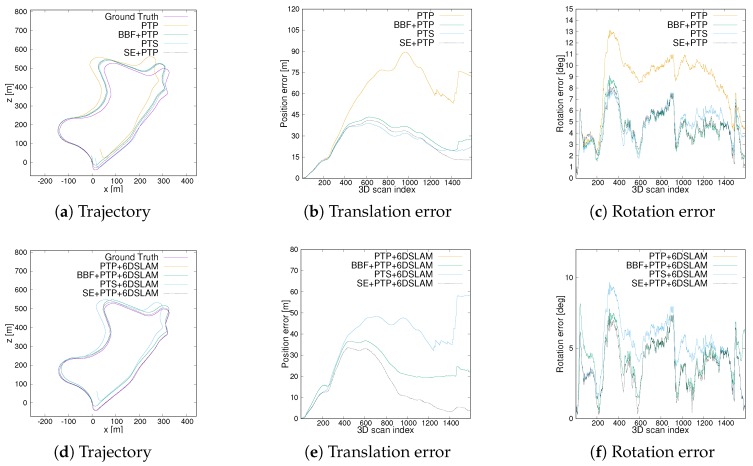
Trajectory and translation as well as rotational error comparison of seq09. (**a**) the trajectory comparison between different ICP. (**b**,**c**) are the translation error and rotational error from ICP. (**d**–**f**) are the corresponding results after applying 6D SLAM.

We also compared these methods in a complex scene mixing urban area and the country. As Figure 13a,b show, the translation accuracy of SE+PTP is inferior to PTS before using 6D SLAM (scan 500 to scan 1200 in Figure 13b). However, it has less rotation error (Table 1) and takes much less time to run than PTS (Table 2). Table 1 shows SE+PTP achieves similar performance to PTS, while PTP suffers from large errors, which Demonstrates PTS performs better in unstructured environments, such as roads and rural areas. In addition, the proposed method can achieve similar performance to PTS after combining segmentation, but it requires less calculation time. In addition, SE+PTP can better close the loop than other methods, cf. Figure 13a.

After 6D SLAM, SE+PTP+6DSLAM is superior to other methods in trajectory error and rotation error, cf. Figure 13e,f. We also find 6DSLAM does improve the accuracy of ICP alone. cf. se09 in Table 1. The reason is 6D SLAM taking into account all scans instead of only two adjacent scans which limits this accumulation error. Although the translation error was reduced from 27.0114 to 18.5825, this error is still rather large, which is caused by the complexity of the scene. Large changes between urban and villages have led to large errors in the middle of this trajectory (scan 300 to scan 800 in Figure 13e). Despite this, our algorithm can still close the loop well, cf. Figure 13d.

The last experiment was conducted in a rural environment, which is a vegetated road and contains little structural information. Please note that this data set is different from the above three groups, because it is a test data set in the KITTI benchmark which only provides the original LiDAR data but does not provide ground truth. To quantitatively analyze the trajectory error, we use the trajectory calculated by the SOFT2 [39] algorithm as the ground truth. SOFT2 is a state-of-the-art stereo visual odometry based on feature selection and tracking. This replacement is reasonable because the accuracy of SOFT2 algorithm is ranked fifth on the KITTI benchmark.

**Figure 14:**
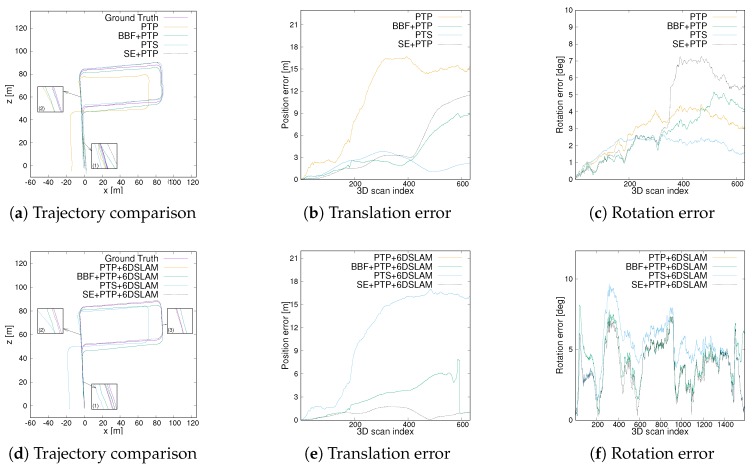
Trajectory and translation as well as rotational error comparison of seq14. (**a**) the trajectory comparison between different ICP. (**b**,**c**) are the translation error and rotational error from ICP. (**d**–**f**) are the corresponding results after applying 6D SLAM.

Figure 14a–c show the performance of SE+PTP is worse than both PTS and BBF+PTP and the gap between the initial position and the end position is larger, cf. Figure 14a(1). However, compared with PTP, SE+PTP reduces the translation error from 12.3578 to 5.5984 (Table 1), and the execution time of the algorithm decreased from 242.4361s to 132.9432 (Table 2). These improvements of performance are mainly due to the introduction of ground point removal and segmentation algorithms. Although PTS achieves higher accuracy before 6D SLAM, it consumes nearly 6 times more time than SE+PTP. The performance of our method has been greatly optimized after 6D SLAM. As shown in Figure 14d(1), the gap between the starting point and the ending point has been largely reduced. Table 1 reports, after 6D SLAM, the translation error was reduced from 5.5984 to 1.0114 while the rotation error is decreased to 0.8563. This shows that our method is superior to similar methods in terms of efficiency and accuracy.

**Figure 15:**
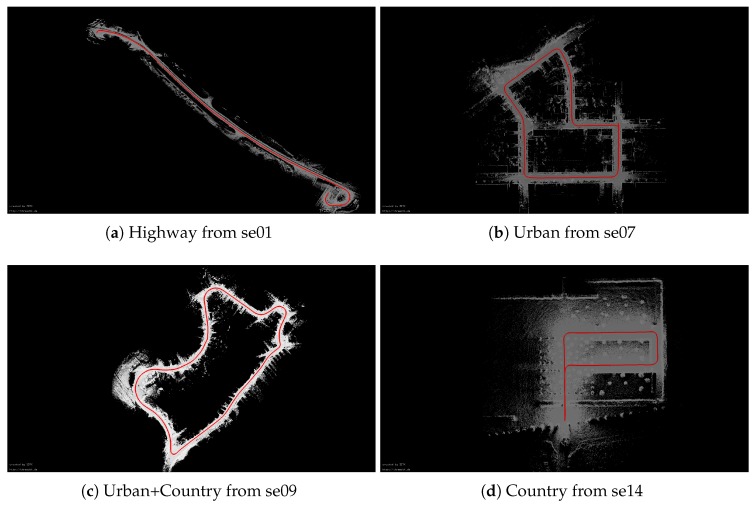
The point-cloud map of four experiments, which is calculated by SE+PTP+6DSLAM. The red line is the trajectory of the vehicle. (**a**) se01. (**b**) se07. (**c**) se09. (**d**) se14.

Figure 15 shows the point-cloud map of four experiments, which is calculated by SE+PTP+6DSLAM. To further test the effectiveness of the proposed algorithm, we evaluate the algorithm using the KITTI metric which calculated the accuracy by averaging relative position and rotation errors using segmented trajectory lengths. The average relative error of all four experiments based on the KITTI metrics is given in Table 3. Please note that only the methods with relatively high accuracy are given here according to Table 1. As shown Table 3, our method achieves higher accuracy. In addition, PTS is slightly inferior to our method in sequences 09 and 14, which demonstrates that point-to-plane ICP performs well in rural areas. This can be attributed to the tangent plane calculated by point-to-plane ICP, which is more robust to unstructured environments. However this also poses a challenge to computing efficiency. As Table 2 shows, PTS consumes nearly 6 times more time than SE+PTP. In sum, the proposed algorithm is superior to the ICP method in both accuracy and efficiency. In addition, our method is more suitable for 6D SLAM.

## 5. Discussion

The core idea of the proposed algorithm is to develop a highly accurate localization and mapping module in unknown environments. We have integrated ground point removal and segmentation modules with the standard point-to-point ICP method. Four experimental results show that both BBF+PTP and SE+PTP greatly improve efficiency and accuracy when compared with the standard ICP method(Table 1). As previously discussed, the LiDAR data contains a large number of ground points, which increase the computational burden as well as the possibility of ICP mismatch. Hence removing ground points is a necessary step. Compared with BBF + PTP, the introduction of the segmentation algorithm leads to higher accuracy of SE+PTP. This is the result that the segmentation algorithm removes those false ground points and noise points. It is worth emphasizing that our method often closes the loop well. After applying 6D SLAM, we also concluded that 6DSLAM is more suitable for optimizing point-to-point ICP, especially for the proposed method.

Our experiments also demonstrated some characteristics about ICP and 6D SLAM. First, the standard point-to-point ICP performs better in urban scene, cf. se07 in Table 1. This is because the environment contains more structured information, such as buildings. However, it has a large error in the country, cf. se09 and se14 in Table 1, while the point-to-plane ICP is more robust to these environments due to the introduction of the tangent plane. Moreover, 6DSLAM does improve the accuracy of point-to-point ICP alone, cf. PTP and PTP+6DSLAM of se09 in Table 1. The reason is 6D SLAM taking into account all scans instead of only two adjacent scans which limits this accumulation error. Although the position accuracy of PTP+6DSLAM in se14 is similar to PTP, the rotation error has been eliminated. However, PTP+6DSLAM shows worse results than PTP in the urban scene (se07 in Table 1). This is because se07 contains a lot of dynamic vehicles which can cause larger error. The performance of standard 6D SLAM degrade in a high dynamic environment. Another issue that must be noted is the performance of PTS+6DSLAM degrades compared to PTS. This problem is caused by the 6DSLAM algorithm itself. Since 6D SLAM is similar to the point-to-point ICP method but taking into account all scans instead of only two adjacent scans. It solves for all poses at the same time and iterates like in the original ICP. Hence, 6D SLAM is more suitable for point-to-point ICP. Furthermore, it must be noted that the point-to-plane ICP method always produces the same result as point-to-point ICP after they are input into 6D SLAM, which is because the 6D SLAM framework is specifically designed for the point-to-point ICP method.

In terms of application scenarios, all methods perform poorly on the highway, which is mainly due to the lack of rich geometric and semantic information on the highway, cf. se01 in Table 1 and Table 3. Due to the lack of semantic information, BBF+PTP finally failed to estimate the pose. This leads to the BBF+PTP-based algorithm to think that the vehicle stayed in place without moving. Hence the scale of this trajectory is reduced by a certain proportion, cf. Figure 9a. In contrast, SE+PTP is more robust, which is mainly due to the introduction of the segmentation algorithm. However, our method still cannot accurately estimate the pose of se01. Because there are too many moving vehicles running with high speed. Although the proposed algorithm perform better than the other methods in se09, it still suffers from large errors due to the complexity of the environment, which is a combination of rural and urban scenes. All methods perform better in the rural environment, i.e., se14, especially the proposed method greatly improves pose accuracy, which is the reason that se14 contains much structural information, e.g., this road is surrounded by trees on both sides and few dynamic objects are contained in this environment. As se14 of Table 1 shows, PTS achieves higher accuracy before 6D SLAM, which is due to it calculates the tangent plane of the point. However, it consumes nearly 6 times more time than SE+PTP, cf. se14 in Table 2. Moreover, the result of SE+PTP+6DSLAM is better than PTS.

Dynamic objects such as high-speed vehicles, are the main error sources affecting pose accuracy. By comparing the locations of errors, we also find that large errors often occur at intersections. As Figure 12 shows, intersections either lack sufficient geometry or contain a large number of dynamic vehicles which are the main cause of errors. In future work, we will carry out research based on dynamic objects removing to further improve the pose estimation accuracy.

## 6. Conclusions

This paper presented a method for enhancing pose estimation accuracy of 3D point clouds by properly processing ground point and point-cloud segmentation. Since the ground points are removed, the proposed method is mainly applied to estimate the pose of ground vehicles. First, a 2D image-based ground point extraction method is introduced as a preprocessing step for ICP matching. Secondly, the point cloud after removing the ground points is then grouped into many clusters. By clustering, some outliers that do not have common attributes are removed. After this step, these different clusters are merged into a new point cloud. Compared to the original point cloud, the ground points of the new point cloud are removed and those false ground points and noise points have also been filtered out, which will greatly increase the efficiency and accuracy of ICP matching. Thirdly, A standard point-to-point ICP is then applied to calculate the six degree-of-freedom transformation between consecutive scans. Once closed loops are detected in the environment, a 6D graph optimization algorithm for global relaxation is employed, which aims to obtain a globally consistent trajectory and mapping.

In addition, we validated the proposed algorithm in four different scenarios including the city, the country and a highway. To test the proposed algorithm, the accuracy and runtime between our method and point-to-point ICP, point-to-plane ICP and Bounding Box Filter-based ICP are presented. Four experimental results show that both BBF+ICP and SE+ICP have improved the accuracy and speed of pose estimation relative to the standard ICP method, demonstrating that removing ground points improve the accuracy, efficiency and robustness of pose estimation based on ground vehicles. Compared with BBF + ICP, the introduction of the segmentation algorithm leads to higher accuracy of SE+ICP. This is the result that the segmentation algorithm removes those false ground points and noise points. Furthermore, we also concluded that 6DSLAM is more suitable for optimizing point-to-point ICP, especially for the proposed method.

In future work, removing dynamic targets of the scene will be fused into this proposed algorithm. Moreover, since our algorithm does not perform well in environments with less geometric information, such as highways, future work will integrate semantic information into our method, which is expected to inevitably improve the efficiency and accuracy of ICP matching. 

## Figures and Tables

**Table 1:** Results of the proposed method (SE+PTP) compared with point-to-point ICP (PTP), BBF+ICP and point-to-plane ICP on the KITTI dataset using absolute metric. *+6D SLAM refers to 6D SLAM is used in the corresponding method. Since sequences 01 does not contain closed loops, 6D SLAM is not employed on sequences. n.a. in this table indicates the corresponding method is not available. *t_err_* represents RMSE(root-mean-square error) of absolute translation error, while *r_err_* represents RMSE of absolute rotation errors.

Method	se01(Highway)		se07(Urban)		se09(Urban+Country)		se14(Country)	
	*t_abs_* [m]	*r_abs_* [deg]	*t_abs_* [m]	*r_abs_* [deg]	*t_abs_* [m]	*r_abs_* [deg]	*t_abs_* [m]	*r_abs_* [deg]
PTP	183.8600	6.7351	7.0136	3.4204	61.6887	8.8175	12.3578	3.1265
BBF+PTP	543.1686	12.0947	6.7612	3.7937	30.5351	4.8372	4.3729	3.0239
PTS	261.8181	22.4791	10.9247	3.6953	**27.0114**	5.0372	**2.3129**	**2.0502**
SE+PTP	**49.0841**	**4.4752**	**3.8886**	**2.7021**	27.4586	**4.7811**	5.5984	4.4430
PTP+6DSLAM	n.a.	n.a.	30.5522	15.9400	39.4745	5.4160	12.6070	1.6986
BBF+PTP+6DSLAM	n.a.	n.a.	8.6707	3.2422	24.7700	4.5936	3.5380	1.6498
PTS+6DSLAM	n.a.	n.a.	30.5522	15.9400	39.4745	5.4160	12.6070	1.6986
SE+PTP+6DSLAM	n.a.	n.a.	**3.0454**	**2.6856**	**18.5825**	**4.1382**	**1.0114**	**0.8563**

**Table 2:** Total program running time of every sequence with different methods. Please note that the time of point-cloud projection, ground point removal and segmentation have been included in SE+PTP. Only the time before using 6D SLAM is given here.

Sequences	Number of Scans	PTP(s)	BBF+PTP(s)	PTS(s)	SE+PTP(s)
se01	1101	534.9202	**235.6870**	1518.7555	351.6523
se07	1101	256.9504	199.3139	962.3334	**191.0013**
se09	1591	592.2633	368.2708	2000.6465	**311.0511**
se14	631	242.4361	183.1523	774.3427	**132.9431**

**Table 3:** Results of the proposed method(SE+PTP) compared with PTS and BBF+PTP+6DSLAM using relative (KITTI metric) metric, where t represents translation error, while r represents rotation error. Since sequences 01 do not contain closed loops, 6D SLAM is not employed on this sequence, hence, for se01, the results before and after 6D SLAM have the same error values.

Method	se01(Highway)		se07(Urban)		se09(Urban+Country)		se14(Country)	
	*t_rel_* [%]	*r_rel_* [deg]	*t_rel_* [%]	*r_rel_* [deg]	*t_rel_* [%]	*r_rel_* [deg]	*t_rel_* [%]	*r_rel_* [deg]
PTS	12.0361	0.0245	3.1390	0.0151	4.2922	0.0198	1.4128	0.0101
SE+PTP	3.9835	0.0080	1.5691	0.0136	4.1093	0.0189	3.0411	0.0301
BBF+PTP+6DSLAM	34.0821	0.0127	2.8382	0.0182	4.4884	0.0201	2.1407	0.0122
SE+PTP+6DSLAM	3.9835	0.0080	1.4075	0.0131	3.9607	0.0183	0.9815	0.0143
